# 30 day mortality in adult palliative radiotherapy – A retrospective population based study of 14,972 treatment episodes

**DOI:** 10.1016/j.radonc.2015.03.023

**Published:** 2015-05

**Authors:** Katie Spencer, Eva Morris, Emma Dugdale, Alexander Newsham, David Sebag-Montefiore, Rob Turner, Geoff Hall, Adrian Crellin

**Affiliations:** a St James’s Institute of Oncology, Leeds Cancer Centre, St James’s University Teaching Hospital, United Kingdom; b Section of Clinical Oncology, Institute of Cancer and Pathology, University of Leeds, St James’s University Teaching Hospital, United Kingdom; c Cancer Epidemiology Group, Section of Epidemiology and Biostatistics, Institute of Cancer and Pathology, University of Leeds, St James’s University Teaching Hospital, United Kingdom

**Keywords:** 30 day mortality, Palliative radiotherapy, Clinical indicator, Fractionation

## Abstract

*Background:* 30-day mortality (30DM) has been suggested as a clinical indicator of the avoidance of harm in palliative radiotherapy within the NHS, but no large-scale population-based studies exist. This large retrospective cohort study aims to investigate the factors that influence 30DM following palliative radiotherapy and consider its value as a clinical indicator.

*Methods:* All radiotherapy episodes delivered in a large UK cancer centre between January 2004 and April 2011 were analysed. Patterns of palliative radiotherapy, 30DM and the variables affecting 30DM were assessed. The impact of these variables was assessed using logistic regression.

*Results:* 14,972 palliative episodes were analysed. 6334 (42.3%) treatments were delivered to bone metastases, 2356 (15 7%) to the chest for lung cancer and 915 (5.7%) to the brain. Median treatment time was 1 day (IQR 1–7). Overall 30DM was 12.3%. Factors having a significant impact upon 30DM were sex, primary diagnosis, treatment site and fractionation schedule (*p* < 0.01).

*Conclusion:* This is the first large-scale description of 30-day mortality for unselected adult palliative radiotherapy treatments. The observed differences in early mortality by fractionation support the use of this measure in assessing clinical decision making in palliative radiotherapy and require further study in other centres and health care systems.

Half of all radiotherapy treatment episodes in England in 2012 were delivered with palliative intent (65,580 episodes) [1]. Palliative radiotherapy is widely used to relieve symptoms from either the primary tumour or sites of metastatic disease in advanced cancer. Clinical trials have demonstrated that hypofractionated treatment provides equivalent symptomatic benefit to longer courses, with limited toxicity [2]. The decision to fractionate treatment, with increased acute toxicity and treatment burden, is sometimes made when it is considered necessary to relieve symptoms or with the aim of durable disease control, although the evidence base for this approach is limited. The balance between symptomatic benefit and the opportunity costs associated with excessive interventions must, therefore, be carefully considered and studied.

Many factors may influence the decision to offer and to fractionate palliative radiotherapy. These include the performance status of the patient, anatomical site of disease, primary diagnosis, co-morbidity, age, access to a clinical oncology opinion, travelling time to the treatment centre, clinician specific factors (including financial incentives) and the estimated life expectancy of the patient [3]. However many of these factors are not prospectively recorded in national datasets.

Studies have shown that oncologists are poor at predicting survival of patients with advanced cancer with a tendency to be overly optimistic [4,5]. This may expose terminally ill patients to the burden of longer fractionated courses of radiotherapy [5,6]. Such overly aggressive cancer care at the end of life has a detrimental effect on quality of life and has previously been suggested as a quality of care issue [7,8]. Conversely, fear of over treatment amongst medical colleagues has also been cited as a possible factor reducing access to palliative radiotherapy [9].

The palliative intent of treatment in patients with symptoms of advanced cancer means it is inevitable that early mortality due to disease progression will occur in some patients. The NHS policy document, ‘Improving outcomes: A strategy for cancer’, proposed mortality within 30-days of treatment (a commonly used metric in other health interventions) as a clinical indicator to assess the avoidance of harm in palliative radiotherapy [10]. Early, US based, studies examining 30-day mortality (30DM) in palliative radiotherapy showed significant mortality in some groups [11,12], but no large population-based studies have been reported. These studies do not consider the relationship between fractionation and outcomes, focussing on access to treatment. Prognostic models for life expectancy amongst the general cancer population [13,14] and specifically death within 30 days of palliative radiotherapy [15] have recently been published. However these are not used in routine clinical practise.

Alongside the need to ensure avoidable harm is minimised, there is a need for global healthcare systems to justify treatments in terms of value for money. Excessive fractionation may be considered in both these contexts (hypofractionation being increasingly advocated in the USA) [16]. Measures which can aid the assessment of the appropriateness of treatment are, therefore, needed.

The use of 30DM as a clinical indicator for the avoidance of harm, through appropriate patient selection, in palliative radiotherapy has not previously been demonstrated. This study investigated the rate of 30DM following palliative radiotherapy in a single cancer centre serving a population of 2.8 million over a 7 year period and considered its value as a clinical indicator.

## Methods

All radiotherapy episodes delivered in a large UK cancer centre (Leeds Cancer Centre), between January 2004 and April 2011, were identified using the electronic patient record system (Patient Pathway Manager (PPM)). PPM collates and prospectively integrates electronic information on all cancer patients treated within the centre; patient (date of birth and sex) and treatment information (date of treatment, planned fractionation, dose, intent of treatment and site of treatment) were extracted for this analysis.

These data were then linked to the cancer registrations held by the National Cancer Registration Service (Northern and Yorkshire) and diagnostic, death and socioeconomic status (SES) information was extracted for all linked records. SES was categorised on the basis of rank quintile of deprivation score (Index of Multiple Deprivation (IMD), ONS 2010 version) [17], for the Lower Super Output Area (population defined geographical region of approximately 1500 people [18]) the patient lived in at diagnosis.

Leeds Cancer Centre (LCC) is a university affiliated centre serving a population of 2.8 million. The number of clinical oncologists increased from 18 to 30 during the study period. All oncologists are site specialised to a maximum of three primary diagnostic groups and are trained in the use of palliative radiotherapy. LCC is resourced through a national NHS tariff system where the reimbursement of the centre reflects the complexity of treatment planning and separately the number of fractions with complexity of treatment delivery. LCC were early adopters of the evidence supporting hypofractionation within palliative radiotherapy. Throughout the study period treatment has been delivered within well-defined clinical protocols e.g., palliative radiotherapy for uncomplicated bone metastases is delivered as a single fraction unless there is clear justification for a fractionated high dose approach. Departmental clinical protocols and a robust electronic patient record allow the study cohort to be defined.

### Definition of palliative intent

Treatment intent was identified as palliative by the treating clinician (centre policy) or if delivered in less than five fractions (exceptions to this were identified e.g., stereotactic body radiotherapy). The site treated was allocated as bone, brain, chest, soft tissue (e.g., treatment to the chest for oesophageal cancer), or unknown on the basis of the treatment site protocol (a free text field entered at the time of treatment), the diagnosis and intention of treatment.

**Fig. 1:**
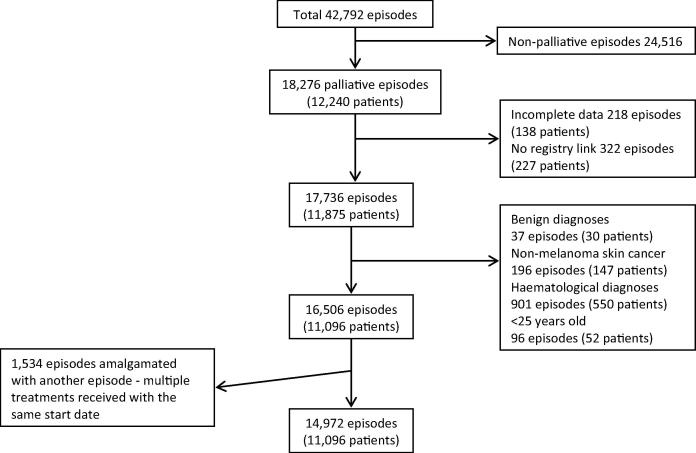
Consort diagram demonstrating exclusions from the study population.

In order to limit this investigation to adult palliative radiotherapy treatments, for solid organ tumours and to ensure data quality, a number of exclusions were made (Fig. 1). Radical treatments (24,516), episodes with incomplete data (540), treatments for benign diagnoses (37), non-melanomatous skin cancer (196) and haematological diagnoses (901) were excluded. Within the centre patients under the age of 25 are treated within the paediatric and young adolescent practice, 96 episodes delivered to this group were also excluded. Where multiple palliative treatments were delivered with the same start date, these were amalgamated into a single record (having been related to a single clinical decision). The fractionation allocated to this event was the largest of the concurrent treatments, this being the more significant clinical decision. 1534 episodes were amalgamated with another record in this way and considered as a single episode. Where overlapping treatment episodes were delivered with differing start dates it is not possible to know if these relate to a single clinical decision. For clarity these were considered separately.

The primary diagnosis was categorised into seven groups based on the most commonly occurring tumours. The major primary diagnoses were lung, breast, prostate, colorectal, bladder and oesophagus, with a separate category, ‘other’, consisting of all other cancer diagnoses and those patients with multiple, non-coincident diagnoses.

### 30-day mortality and survival

The proportion dying within 30-days from treatment start was assessed for all treatments within the cohort and by numbered courses in relation to fractionation delivered, primary diagnosis and site treated. The Chi-squared test was used to assess the impact of various factors upon early mortality. A logistic regression model was used to investigate the factors associated with death within 30-days of the start of palliative radiotherapy. The dependent variable, death within 30-days, was considered as a binary outcome. Covariates (explanatory variables) in the model included, age at start of radiotherapy, sex, socioeconomic status, site of the primary tumour, site of irradiation, fractionation pattern and year of treatment.

Survival was calculated from the start of each palliative radiotherapy episode to date of death or when censored (30th April 2012). The start date of treatment was used as it is closer to the clinical decision to treat than the end of treatment and provides a uniform time point across all fractionation regimens, aligning with NCEPOD systemic therapy methodology [19]. As individuals who underwent multiple sequential treatment episodes had, by definition, to survive all previous treatments and to ensure people could not enter survival analyses twice the univariate logistic regression model and illustrative Kaplan–Meier survival curves were produced based on first and second treatment episodes separately. Multivariate analysis considered only the first treatment episode. Univariate logistic regression was also carried out for all treatment episodes combined, this overall analysis is likely to be a closer reflection of the measure as applied in future, on a population level; including every clinical decision within the cohort. Statistical analyses were carried out using STATA IC 13.

## Results

42,792 radiotherapy treatment episodes were identified. Within this a total of 18,275 palliative treatment episodes, delivered to 12,240 individuals, were identified. Of these, 3303 (18.1%) episodes in 1144 individuals were excluded (Fig. 1), leaving a study population of 14,972 episodes delivered to 11,096 people.

Table 1 shows the characteristics of the population undergoing palliative radiotherapy. The median age at treatment was 70 years (range 25–101). The majority of palliative radiotherapy episodes (55.3%) were delivered to men. Lung (25.3%), breast (14.7%), prostate (14.5%) and colorectal (5.2%) cancers were the most frequently treated primary diagnoses. The commonest irradiated site was bone with 4407 individuals receiving 6334 (42.3%) courses of treatment to bone alone or bone combined with another site. Soft tissue (3691 (24.7%)) and chest (3628 (24.2%)) were the next most commonly irradiated sites.

61.9% of patients received treatment consisting of four or less radiotherapy fractions (50.5% single and 11.4% 2–4 fractions). 23.7% of patients received five fractions, 2.2% received 6–9 treatments and 12.3% 10 or more fractions.

### 30-day mortality and survival

**Supplementary Fig. 1S:**
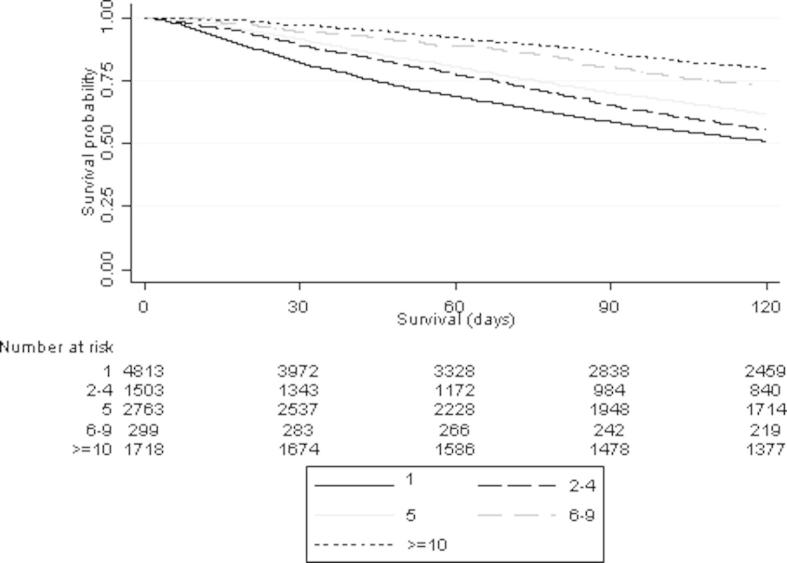
Survival following the start of palliative radiotherapy in relation to fractionation pattern.

**Fig. 2:**
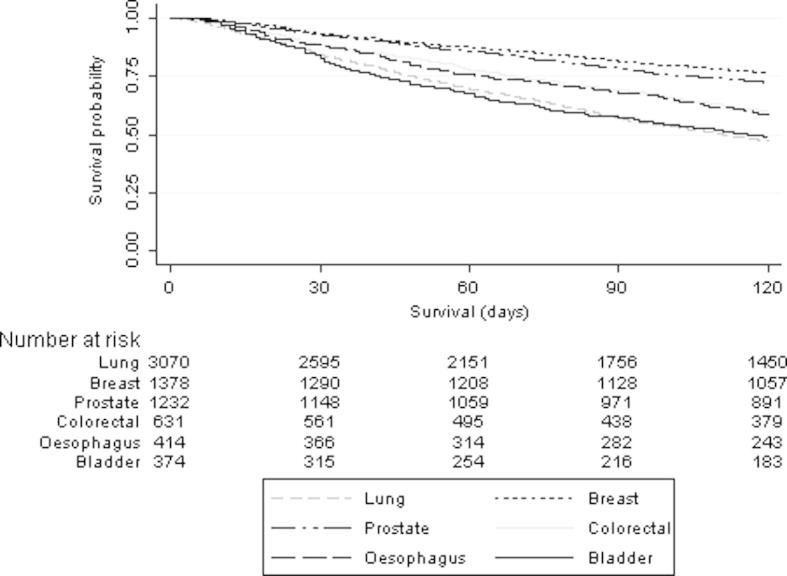
Survival and 30 DM following the start of palliative radiotherapy in relation to primary diagnosis.

Overall, 1846 (12.3%) individuals died within 30 days of the initiation of a course of palliative radiotherapy. Variations of 30DM in relation to the characteristics of the population and treatment episodes are shown in Table 2. The median survival time for the whole cohort was 169 days (Inter-Quartile Range (IQR) 67–436 days). Significant variation in survival patterns was seen however in relation to both the primary diagnosis and fractionation pattern (Fig. 2 and Supplementary Fig. 1S respectively).

**Supplementary Fig. 2S:**
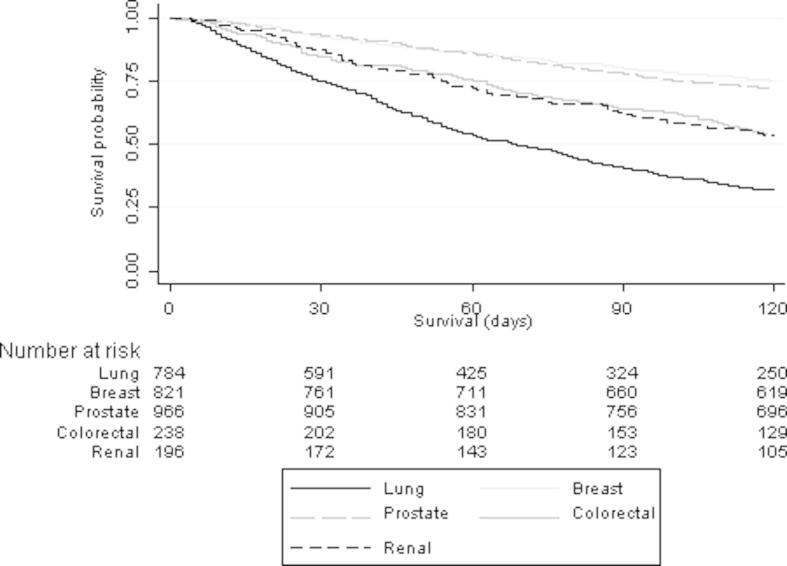
Survival following palliative radiotherapy to bone by primary diagnosis.

**Fig. 3:**
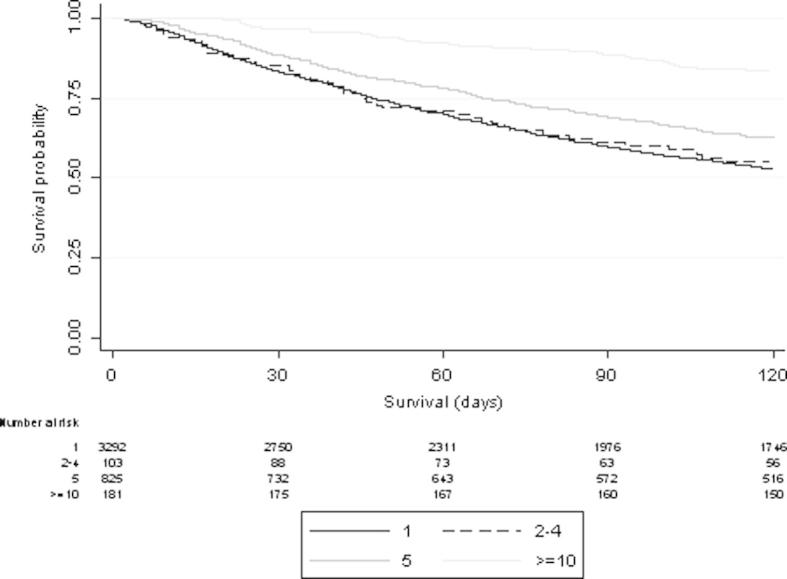
Survival and 30 DM following palliative radiotherapy to bone by fractionation pattern (6–9 fraction treatments are not included here due to small numbers (*n* = 6)).

Table 3, Fig. 3 and Supplementary Fig. 2S show patterns of radiotherapy fractionation, 30DM and survival following treatment to bone. Overall 30DM was 14.1%; there was significant variation in relation to primary diagnosis and fractionation pattern (*p* < 0.001).

Palliative radiotherapy to the chest for lung cancer accounted for 2356 (15.7%) treatments and was associated with 30DM of 14.0%, this was significantly related to fractionation, *p* < 0.01. Median treatment time for all palliative treatments for lung cancer to the chest was 8 days (IQR 1–12). The most commonly used fractionation schemes reflect local protocols: 1 (32%), 2 (30%), 5 (13.72%), 12 (8.64%) and 13 (7.70%).

**Supplementary Fig. 3S:**
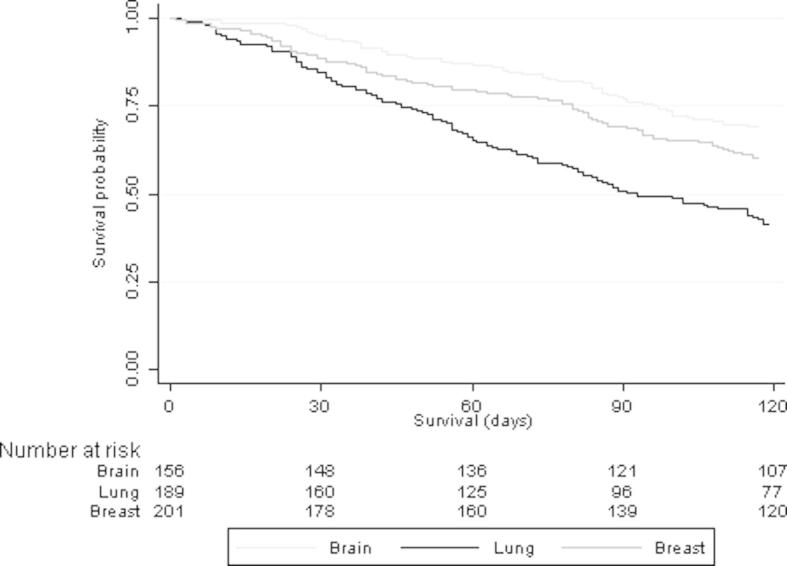
Survival following palliative radiotherapy to the brain by primary diagnosis.

915 (5.65%) episodes of palliative radiotherapy to the brain were delivered within the cohort, of these 68 were accompanied by treatment to a second site. The two most frequently treated metastatic diagnoses were breast and lung cancer (205 (22.4%) and 192 (21.0%) respectively of total) with primary brain tumours accounting for 160 (17.5%) treatments. Overall 30DM was 11.2%, with breast, lung, and primary brain cancers having 30DM of 11.2%, 15.1%, and 5.6% respectively (Supplementary Material, Fig. 3S).

Significant variation in 30DM was apparent in relation to sex, age, primary diagnosis, treatment site, IMD and fractionation schedule adopted. Age and IMD did not retain their significance in multivariate analysis (Supplementary Material Table 3S). Socioeconomic status (SES) (as measured by IMD) has been shown to impact significantly upon cancer outcomes [20], the reasons for this are not entirely clear however SES may be a surrogate for co-morbidity. Females had a 16% reduction in the odds of death within 30 days compared to males (Odds Ratio (OR) 0.84, 95% Confidence Interval (CI) 0.74–0.96, *p* = 0.010) even after adjustment for other case mix factors. A statistically significant relationship between increasing fractionation schedules and reduction in the odds of death within 30-days was observed. Those receiving 10 or more fractions were 90% less likely to die within 30-days of the start of radiotherapy compared to those receiving just one fraction (OR 0.10, 95% CI 0.08–0.14, *p* < 0.001). Of note there was no significant variation in early mortality with time (*p* = 0.391).

## Discussion

This is the first large, population-based study investigating 30DM following palliative radiotherapy in a single centre that is the sole provider of radiotherapy to a large population. An overall 30 DM rate of 12.3% was observed, aligning well with other recently published data for first palliative radiotherapy treatments [13].

In this cohort of patients the site most frequently irradiated was bone. Overall 30DM for these treatments was 14.1%. The literature suggests the median time to treatment benefit following palliative radiotherapy to bone is 14 days, with response rates of between 50% and 70% [21,22]. The risk of pathological fracture and the need for re-treatment, following single fraction radiotherapy to bone metastases may contribute to the use of fractionated radiotherapy in this setting. Median time to re-treatment has been reported to be 25 weeks after a single fraction [23]. International evidence strongly favours the use of single fraction treatments in uncomplicated bone metastases [21,24]. Our early mortality outcomes suggest this evidence base has been appropriately applied in this study population. Fractionated courses of radiotherapy to bone were associated with significantly lower 30DM.

Palliative radiotherapy to the brain had a 30DM of 11.6% overall. Despite the selective use (⩽40 episodes, ⩽10 deaths) of palliative radiotherapy to the brain for some poor prognosis groups within our cohort, early mortality remains high (e.g., melanoma (21.9%), unknown primary (24.0%) (data not shown)). The benefit of whole brain radiotherapy for metastases, as compared to supportive care alone, has not been assessed in randomised controlled trials in the CT era [25], the final outcome of the QUARTZ trial is awaited [26].

30DM for lung cancer patients treated with palliative radiotherapy was 17.3% (similar to equivalent figures published in the USA [12]). Those receiving treatment to the primary had a 30DM of 14.1%, which was significantly related to fractionation (*p* < 0.01). 30DM following 1–2 fraction treatments (22.5%) was markedly higher than that following ten or more fraction treatments (2.5%). Median time to symptom improvement following thoracic radiotherapy for NSCLC is approximately 1–2 months [27,28]. Excessive fractionation in poor performance status, lung cancer patients cannot be justified given well documented evidence supporting the use of hypofractionation for equivalent symptom control in this group [28].

30DM varies significantly with primary diagnosis, the site being irradiated, patient sex and the fractionation pattern chosen. Greater use of more fractionated treatments, increased treatment burden and potentially higher acute toxicity are harder to justify in diagnoses with poor prognosis. There are a limited number of palliative settings in which more fractionated treatments are known to improve survival. We believe that the variation demonstrated with fractionation reflects appropriate clinical decision making, encompassing predicted prognosis, in the context of clinical protocols which in themselves reflect the underpinning evidence. This supports the use of 30DM as a measure of clinical decision making in palliative radiotherapy.

Survival with symptomatic disease has increased with increasing systemic therapy options. It is unclear however if the point within the disease trajectory at which palliative radiotherapy is delivered has changed. Lack of variation in early mortality with time demonstrated here suggests that clinical decision making near the end of life is stable.

We have shown that it is possible to audit 30DM, in a large unselected population. The size of this dataset allows analysis of subgroups by diagnosis and site treated whilst also maintaining statistical power. In addition, the single centre from which the data were derived was an early adopter of the evidence supporting the hypofractionation of palliative radiotherapy (increasingly advocated globally, including in the USA) [16] and patients were treated within site specific teams, to consistent clinical protocols (adopted at the start of the study period). This study has some limitations:

• It reflects practice within a single, large NHS centre.
• Planned fractionation was used for all analyses as it most closely reflects the clinical decision to treat, however it may not always be the fractionation actually delivered. Delivered fractionation was not available across the whole cohort. If, in the future, such data can be captured the strength of any analyses undertaken would be increased.
• Data within the electronic patient record reflect routine clinical practice and were not prospectively coded for research purposes. The allocation of target tissue and intent of treatment was based upon fixed algorithms. Manual review, by investigating clinicians, of a sample of cases, revealed high levels of concordance of these fields (results not shown) indicating allocation algorithms were robust. A database with defined coding rules, to include not only anatomical site but also target tissue and re-irradiation would also be beneficial. Whilst the current national Radiotherapy Dataset (RTDS) includes coding of anatomical site these data are inadequate due to variations in the application of the coding rules.

It has previously been shown that chemotherapy prescribing behaviour among medical oncologists can be influenced by feeding back early mortality outcomes [5,7,29,30]. Oncologists prescribing palliative radiotherapy often do not have an opportunity to follow up individual patients, so providing feedback in the form of 30DM outcomes for palliative radiotherapy to clinicians or teams may be beneficial. A future analysis of 30DM within the RTDS would also allow comparisons between centres and may be of value in clinical policy setting and commissioning. Outlying results would merit further study to determine the underlying causes and whether this reflected appropriate variation in practise, refinement of this assessment process is anticipated over time.

NCEPOD [19] in surgery and chemotherapy has led to the routine practise of a retrospective review of all deaths within 30 days. We would recommend an analogous approach, however for single fraction palliative radiotherapy this may be impractical due to the high numbers (16.7%). In those receiving a single fraction the burden of treatment is minimal but the potential for benefit within 30 days still significant. An earlier time point, possibly mortality within 14 days (suggested from the survival curves in Fig. 2S (Supplementary Material)), may be more pragmatic for a retrospective case note review. The variation in treatment burden and incremental benefits with fractionation must be borne in mind when considering 30DM outcomes. Whilst many factors may contribute to this decision making process clinicians must be vigilant to the risk of early mortality when deciding to fractionate palliative treatments. Practise will vary, with evidence demonstrating the impact of financial incentives upon the fractionation of radiotherapy [3]. These may be in direct conflict with the need to reduce treatment burden for terminally ill patients. As the availability of highly conformal radiotherapy increases, the use of high dose, hypofractionated palliative radiotherapy will increase. The benefits in terms of reduced treatment burden (both from reduced normal tissue toxicity and decreased visits) are clear, however the cost implications for the department are significant and careful case selection will remain important.

30DM has been recommended as a clinical indicator of the avoidance of harm in palliative radiotherapy. It is suited to this in a number of ways: It is objective, clinically relevant, measurable in a timely manner at a population level and may encourage improvements both in the avoidance of harm and cost-effectiveness of palliative radiotherapy services. However, there are limitations to the use of 30DM. It is a single outcome measure; patient reported outcomes of symptomatic benefit and re-irradiation rates would be valuable as complimentary measures to provide reassurance that patients were not being undertreated. More fractionated palliative radiotherapy would be expected to have a lower 30DM but by contrast any move to reduce 30DM without reference to fractionation patterns may have a detrimental impact on access to appropriate hypofractionated palliative radiotherapy. There will be considerable debate about what constitutes optimal 30DM. Future work, involving more in depth analysis of a smaller population, allowing assessment of performance status, patient preferences and outcomes would be valuable. This, alongside an assessment of the impact of implementing 30DM, would allow validation of the measure as a clinical indicator.

This is the first large-scale description of 30-day mortality for unselected adult palliative radiotherapy treatments. Significant variation is demonstrated with diagnosis, sex, treatment site and, importantly, fractionation. In this setting, a measure which can help to assess the appropriateness of treatment and avoidance of harm (as demanded by providers of health care) [10] is required. 30DM has a significant value as a retrospective measure of departmental palliative radiotherapy outcomes when considered alongside fractionation patterns. Clearly separated 30DM outcomes by fractionation would provide reassurance that clinical decision making was appropriate. It does not attempt to assess or guide individual clinical decisions. The observed differences in early mortality by fractionation support further study in other centres and health care systems. Our results suggest it is of value in assessing department wide clinical decision making in palliative radiotherapy providing parity with the early metrics used in other healthcare interventions.

## Authors contributions

K.S., E.M., E.D., A.N., D.S.M., R.T., A.C. and G.H. were all involved in the study design and interpretation of results. A.N. was responsible for data extraction and linkage. E.M. and K.S. were responsible for statistical analyses. E.M. was responsible for production of figures. K.S., E.D. and A.C. conducted the literature review. K.S., A.C., E.M., R.T., G.H., E.D. and D.S.M. were involved in preparation of the manuscript. A.N. reviewed the manuscript.

## Declaration of interests

Dr. Crellin reports salary support from Department of Health/NHS, England National Clinical Lead for Proton Beam Therapy, outside the submitted work; and Co-Chair of the National Radiotherapy Implementation Group (NRIG) 2012–2013.

## Figures and Tables

**Table 1:** Characteristics of the study population. The majority of the population underwent a single episode of palliative radiotherapy, but 2625 individuals underwent two or more episodes (3876 episodes). Only 772 patients received three or more courses. Due to the limited size of this latter population information is presented by first episode, second episode and overall within the cohort.

Characteristic		Episode number				All episodes	
		1		2			
		*n*	%	*n*	%	*n*	%
Age at initial palliative radiotherapy	⩽50	847	7.6	275	10.5	1273	8.5
	51–60	1766	15.9	490	18.7	2529	17.0
	61–70	3051	27.5	769	29.3	4197	28.2
	71–80	3439	31.0	736	28.0	4503	30.2
	>80	1993	18.0	355	13.5	2470	16.6

Sex	Male	6053	54.6	1478	56.3	8244	55.3
	Female	5042	45.4	1147	43.7	6727	45.1

IMD category	Most deprived	2663	24.0	574	21.9	3501	23.5
	2	2194	19.8	515	19.6	2955	19.8
	3	1849	16.7	425	16.2	2466	16.6
	4	2318	20.9	565	21.5	3157	21.2
	Most affluent	1841	16.6	504	19.2	2606	17.5
	Unknown	231	2.1	42	1.6	287	1.9

Primary cancer diagnosis	Other	3997	36.0	852	32.5	5185	34.8
	Lung	3070	27.7	548	20.9	3770	25.3
	Breast	1378	12.4	478	18.2	2186	14.7
	Prostate	1232	11.1	533	20.3	2154	14.5
	Colorectal	631	5.7	120	4.6	778	5.2
	Oesophagus	414	3.7	51	1.9	468	3.1
	Bladder	374	3.4	44	1.7	431	2.9

Site of irradiation	Multiple	892	8.0	285	10.9	1339	9.0
	Bone	3321	29.9	1294	49.3	5379	36.1
	Brain	704	6.3	106	4.0	846	5.7
	Chest	3224	29.1	337	12.8	3628	24.3
	Soft tissue	2901	26.1	576	21.9	3691	24.8
	Unknown	54	0.5	27	1.0	89	0.6

Fractionation	1	4813	43.4	1759	67.0	7558	50.5
	2–4	1503	13.5	157	6.0	1706	11.4
	5	1763	15.9	585	22.3	3547	23.7
	6–9	299	2.7	24	0.9	325	2.2
	⩾10	1718	15.5	100	3.8	1836	12.3

Year of treatment	2004	1636	14.7	272	10.4	1982	13.2
	2005	1489	13.4	386	14.7	2071	13.8
	2006	1430	12.9	362	13.8	1990	13.3
	2007	1345	12.1	340	13.0	1847	12.3
	2008	1535	13.8	330	12.6	2024	13.5
	2009	1498	13.5	399	15.2	2086	13.9
	2010	1666	15.0	398	15.2	2274	15.2
	2011	497	4.5	138	5.3	698	4.7

Total		11,096	74.1	2625	17.5	14,972	100.0

**Table 2:** 30-day mortality in relation to the characteristics of the population (as in Table 1, episodes are considered as first, second and overall within the cohort).

Characteristic		Episode 1				Episode 2				All episodes			
		*n*	Deaths within 30 days		*P* value	*n*	Deaths within 30 days		*P* value	*n*	Deaths within 30 days		*P* value
			*n*	%			*n*	%			*n*	%	
Age at initial palliative radiotherapy	⩽50	847	85	10.04	0.110	275	40	14.55	0.084	1273	141	11.08	0.010
	51–60	1766	212	12.00		490	70	14.29		2529	327	12.93	
	61–70	3051	382	12.52		769	116	15.08		4197	547	13.03	
	71–80	3439	438	12.74		736	92	12.50		4503	573	12.72	
	>80	1993	219	10.99		355	33	9.30		2470	258	10.45	

Sex	Male	6053	795	13.13	<0.001	1478	215	14.55	0.045	8244	1110	13.46	<0.001
	Female	5042	541	10.73		1147	136	11.86		6727	736	10.94	

IMD category	Most deprived	2663	332	12.47	0.148	574	87	15.16	0.079	3501	459	13.11	0.025
	4	2194	283	12.90		515	82	15.92		2955	390	13.20	
	3	1849	233	12.60		425	53	12.47		2466	313	12.69	
	2	2318	267	11.52		565	65	11.50		3157	367	11.62	
	Most affluent	1841	191	10.37		504	56	11.11		2606	278	10.67	
	Unknown	231	30	12.99		42	8	19.05		287	39	13.59	

Site of primary	Multiple and Other	3997	479	11.98	<0.001	852	108	12.68	<0.001	4542	643	14.16	<0.001
	Lung	3070	489	15.93		548	133	24.27		3770	652	17.29	
	Breast	1378	92	6.68		478	32	6.69		2186	152	6.95	
	Prostate	1232	89	7.22		533	41	7.69		2154	161	7.47	
	Colorectal	631	72	11.41		120	22	18.33		778	99	12.72	
	Oesophagus	414	50	12.08		50	6	12.00		468	59	12.61	
	Bladder	374	65	17.38		44	9	20.45		431	80	18.56	

Site of irradiation	Multiple	892	173	19.39	<0.001	285	46	16.14	<0.001	1339	242	18.07	<0.001
	Bone	3321	460	13.85		1294	163	12.60		5379	715	13.29	
	Brain	704	71	10.09		106	19	17.92		846	95	11.23	
	Chest	3224	381	11.82		337	73	21.66		3628	467	12.87	
	Soft tissue	2901	241	8.31		576	46	7.99		3691	313	8.48	
	Unknown	54	10	18.52		27	4	14.81		89	14	15.73	

Fraction group	1	4813	873	18.14	<0.001	1759	259	14.72	<0.001	7558	1265	16.74	<0.001
	2–4	1503	166	11.04		157	31	19.75		1706	203	11.90	
	5	1763	235	13.33		585	54	9.23		3547	309	8.71	
	6–9	299	16	5.35		24	2	8.33		325	18	5.54	
	⩾10	1718	46	2.68		100	5	5.00		1836	51	2.78	

Year of treatment	2004	1636	187	11.43	0.745	272	35	12.87	0.865	1982	232	11.71	0.719
	2005	1489	197	13.23		386	51	13.21		2071	278	13.42	
	2006	1430	178	12.45		362	56	15.47		1990	258	12.96	
	2007	1345	161	11.97		340	40	11.76		1847	224	12.13	
	2008	1535	181	11.79		330	42	12.73		2024	240	11.86	
	2009	1498	186	12.42		399	50	12.53		2086	257	12.32	
	2010	1666	186	11.16		398	59	14.82		2274	273	12.01	
	2011	497	60	12.07		138	16	11.59		698	84	12.03	

Total		11,096	1336	12.04		2625	351	13.37		14,972	1846	12.33	

**Table 3:** 30-day mortality following palliative radiotherapy to bone.

Characteristic		Episode 1			Episode 2			All episodes		
		*n*	Deaths within 30 days		*n*	Deaths within 30 days		*n*	Deaths within 30 days	
			*n*	%		*n*	%		*n*	%
Fraction group	1	3292	558	17.0	1005	123	12.2	4863	744	15.3
	2–4	103	15	14.6	26	1	3.8	145	19	13.1
	5	825	95	11.5	198	22	11.1	1117	125	11.2
	6–9	6	0	0.0	2	0	0.0	9	0	0.0
	⩾10	181	3	1.7	12	0	0.0	200	6	3.0

Primary diagnosis	Breast	821	62	7.6	284	13	4.6	1293	90	7.0
	Colorectal	238	36	15.1	40	8	20.0	285	46	16.1
	Lung	784	197	25.1	153	33	21.6	979	237	24.2
	Prostate	966	64	6.6	419	39	9.3	1664	121	7.3
	Renal	196	25	12.8	51	7	13.7	287	40	13.9

Site of irradiation	Bone	3734	532	14.2	1066	123	11.5	5379	715	13.3
	Multiple bony sites	538	113	21.0	143	18	12.6	779	144	18.5
	Bone and another site	135	29	21.5	34	5	14.7	176	35	19.9

Total		4407	674	15.3	1243	146	11.7	6334	894	14.1

## Appendix A Supplementary data

Supplementary Table 1 Multivariate analysis investigating the odds of death within 30-days of the start of radiotherapy (only first treatment episodes were included in this analysis).
