# Supplementary material for: 30 day mortality in adult palliative radiotherapy – A retrospective population based study of 14,972 treatment episodes
Source: Radiother Oncol. 2015 May;115(2):264–71. doi: 10.1016/j.radonc.2015.03.023 (PMC4504022; doi:10.1016/j.radonc.2015.03.023)
Supplement: Supplementary Table 1 — Multivariate analysis investigating the odds of death within 30-days of the start of radiotherapy (only first treatment episodes were included in this analysis). [file mmc1.docx]

| Characteristic | | OR | Lower 95%CI | Upper 95%CI | P value |
| --- | --- | --- | --- | --- | --- |
| Age at start of radiotherapy (per year) | | 1.00 | 0.99 | 1.00 | 0.717 |
| Sex | Male | 1.000 |  |  |  |
|  | Female | 0.841 | 0.737 | 0.959 | 0.010 |
| IMD category | Most deprived | 1.000 |  |  |  |
|  | 4 | 1.053 | 0.883 | 1.255 | 0.567 |
|  | 3 | 1.045 | 0.868 | 1.258 | 0.640 |
|  | 2 | 0.963 | 0.806 | 1.151 | 0.678 |
|  | Most affluent | 0.826 | 0.679 | 1.006 | 0.057 |
|  | Unknown | 0.983 | 0.651 | 1.485 | 0.935 |
| Fraction group | 1 | 1.000 |  |  |  |
|  | 2 to 4 | 0.413 | 0.338 | 0.504 | <0.001 |
|  | 5 | 0.364 | 0.308 | 0.431 | <0.001 |
|  | 6 to 9 | 0.168 | 0.098 | 0.286 | <0.001 |
|  | ≥10 | 0.103 | 0.076 | 0.141 | <0.001 |
| Primary cancer site | Multiple & other | 1.000 |  |  |  |
|  | Lung | 1.361 | 1.159 | 1.597 | <0.001 |
|  | Breast | 0.470 | 0.365 | 0.605 | <0.001 |
|  | Prostate | 0.330 | 0.257 | 0.423 | <0.001 |
|  | Colorectal | 0.842 | 0.642 | 1.104 | 0.214 |
|  | Oesophagus | 1.340 | 0.955 | 1.882 | 0.091 |
|  | Bladder | 1.538 | 1.141 | 2.072 | 0.005 |
| Site of irradiation | Multiple | 1.000 |  |  |  |
|  | Bone | 0.702 | 0.573 | 0.859 | <0.001 |
|  | Brain | 1.109 | 0.800 | 1.538 | 0.535 |
|  | Chest | 0.664 | 0.532 | 0.828 | <0.001 |
|  | Soft tissue | 0.569 | 0.452 | 0.717 | <0.001 |
|  | Unknown | 1.544 | 0.737 | 3.234 | 0.249 |
| Year of radiotherapy | | 0.988 | 0.961 | 1.016 | 0.391 |

Table 1s: Multivariate analysis investigating the odds of death within 30-days of start of radiotherapy (only first treatment episodes were included in this analysis)
